# Enhanced Ohmyungsamycin A Production via Adenylation Domain Engineering and Optimization of Culture Conditions

**DOI:** 10.3389/fmicb.2021.626881

**Published:** 2021-02-17

**Authors:** Eunji Kim, Young Eun Du, Yeon Hee Ban, Yern-Hyerk Shin, Dong-Chan Oh, Yeo Joon Yoon

**Affiliations:** Natural Products Research Institute, College of Pharmacy, Seoul National University, Seoul, South Korea

**Keywords:** ohmyungsamycin, non-ribosomal peptide synthetase, adenylation domain engineering, site-directed mutagenesis, culture condition optimization

## Abstract

Ohmyungsamycins (OMSs) A and B are cyclic depsipeptides produced by marine *Streptomyces* strains, which are synthesized by a non-ribosomal peptide synthetase. Notably, OMS A exhibits more potent activity against *Mycobacterium tuberculosis* and human cancer cells than OMS B. The substrate promiscuous adenylation (A) domain in the second module of OMS synthetase recruits either L-Val or L-Ile to synthesize OMSs A and B, respectively. Engineering of the substrate-coding residues of this A domain increased OMS A production by 1.2-fold, coupled with a drastic decrease in OMS B production. Furthermore, the culture conditions (sea salt concentration, inoculum size, and the supply of amino acids to serve as building blocks for OMS) were optimized for OMS production in the wild-type strain. Finally, cultivation of the A2-domain-engineered strain under the optimized culture conditions resulted in up to 3.8-fold increases in OMS A yields and an 8.4-fold decrease in OMS B production compared to the wild-type strain under the initial culture conditions.

## Introduction

**FIGURE 1:**
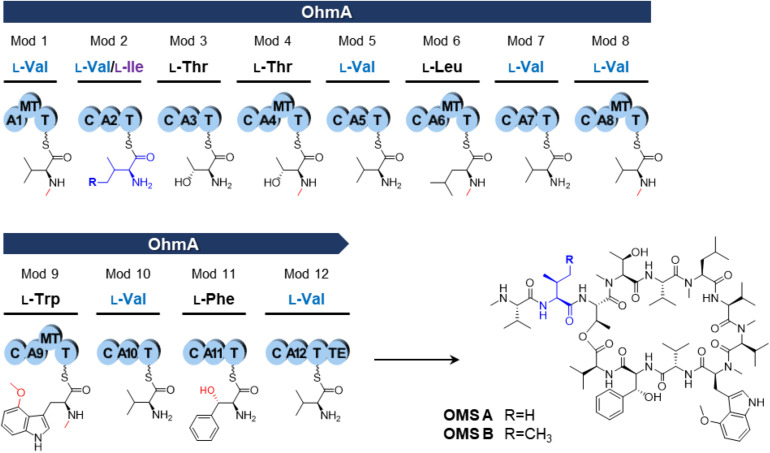
NRPS assembly line of the OMS synthetase enzyme (OhmA) and structures of OMS A and OMS B. Functional groups by modification reaction to each amino acid are shown in red. A, adenylation domain; C, condensation domain; MT, N-methyltransferase domain; T, thiolation domain [also known as peptidyl carrier protein (PCP) domain]; TE, thioesterase domain.

Ohmyungsamycins (OMSs) are macrocyclic peptides with antibacterial and anticancer properties, which are produced by marine bacterial strains belonging to the *Streptomyces* genus (Um et al., 2013; Kim et al., 2017; Byun et al., 2020). OMS A is produced along with its analog, OMS B, for which the chemical structure was verified via chemical synthesis (Hur et al., 2018). However, OMS A is notably more active than OMS B against a diverse range of bacteria and cancer cells (Um et al., 2013; Kim et al., 2017; Byun et al., 2020). Naturally occurring OMSs A and B possess different dipeptide side chains appended to an identical cyclic core peptide (Figure 1). A recent study (Kim et al., 2019) reported that OMSs are biosynthesized by the OhmA encoding non-ribosomal peptide synthetase (NRPS), which is comprised of 12 modules (Figure 1). During NRP synthesis, an adenylation (A) domain generally selects an amino acid substrate and attaches to the adjacent peptidyl carrier protein (PCP) domain and a condensation (C) domain catalyzes peptide bond formation in the growing peptide (Schwarzer et al., 2003). During the OMS biosynthesis process, the second A (A2) domain in module 2, which exhibits relaxed substrate selectivity, activates both L-Val and L-Ile to produce OMS A and OMS B, respectively. Multiple sequence alignments of Val-activating A domains from OMS and other NRPSs show differences in the key residues of the substrate binding pockets that determine substrate specificity (Kim et al., 2019). According to the numbering of amino acid sequence of the GrsA of which crystal structure was determined (Conti et al., 1997; Stachelhaus et al., 1999), the Val-activating A domains of OhmA NRPS were found to possess Trp and Gly residues at positions 299 and 322, respectively, whereas the OhmA-A2 domain possesses Gly and Ala residue substitutions at the aforementioned positions (Table 1). This variation in the key substrate specificity-determining residues of the A2 domain results in its unusual substrate flexibility and suggests that substrate preference may be altered by changing these specific residues. The substrate specificity of A domain in NRPS can be engineered by either site-directed mutagenesis of the specificity-conferring residues or substitution of the A domain. In general, changing individual amino acids through site-directed mutagenesis is less likely to affect the overall structure and functional integrity of NRPS (Kim et al., 2015; Winn et al., 2016).

**TABLE 1:** Amino acid residues of substrate binding pockets in the Val-activating A domains from OMS and other NRPS: Ohmyungsamycin synthetase A (OhmA), surfactin synthetase B (SrfAB), gramicidin S synthetase B (GrsB), and tyrocidine A synthetase C (TycC).

A domain	Substrate specific residue (PheA numbering) [a]									
	235	236	239	278	299	301	322	330	331	517
OhmA-A1	D	A	Y	W	W	G	G	T	F	K
OhmA-A2	D	A	Y	W	G	G	A	T	F	K
OhmA-A5	D	A	Y	W	W	G	G	T	F	K
OhmA-A7	D	A	Y	W	W	G	G	T	F	K
OhmA-A8	D	A	Y	W	W	G	G	T	F	K
OhmA-A10	D	A	Y	W	W	G	G	T	F	K
OhmA-A12	D	A	Y	W	W	G	G	T	F	K
SrfAB-A4	D	A	F	W	I	G	G	T	F	K
GrsB-A3	D	A	F	W	I	G	G	T	F	K
TycC-A8	D	A	F	W	I	G	G	T	F	K

*[a] The numbering corresponds to the residues of the PheA domain of GrsA crystal structure.*

Therefore, to effectively produce the more desirable compound OMS A, our study engineered the A2 domain by altering the aforementioned specificity-conferring residues using site-directed mutagenesis. Additionally, culture conditions were optimized for OMS A production in the wild-type OMS-producing strain. Cultivation of the engineered strain in the optimized conditions led to a significant increase in OMS A production with only trace amounts of OMS B.

## Materials and Methods

### Bacterial Strains, Plasmids, and Culture Conditions

Table 2 summarizes the bacterial strains and plasmids used in this study. The OMS-producing strain *Streptomyces* sp. SNJ042 was isolated from a sediment sample obtained from Jeju Island in Korea (Um et al., 2013). The wild-type SNJ042 strain and its derivative mutant strains were propagated on ISP4 medium (Shirling and Gottlieb, 1966) at 30°C. *Escherichia coli* DH5α was used as the host for general cloning, and non-methylating *E. coli* ET12567/pUZ8002 was used for conjugal transfer of recombinant plasmids between *E. coli* and *Streptomyces* (Kieser et al., 2000). DNA fragments for plasmid construction were obtained from SNJ042 genomic DNA via polymerase chain reaction (PCR) using GXL DNA polymerase (Takara, Shiga, Japan) according to the manufacturer’s recommended conditions. The pGEM T-Easy vector (Promega, Madison, WI, United States) was used for subcloning and the temperature-sensitive *E. coli*–*Streptomyces* shuttle vector pKC1139 (Bierman et al., 1992) was used for gene recombination.

**TABLE 2:** Bacterial strains and plasmids used in this study.

Strain and plasmid	Description	References
**Strain**		
*E. coli* DH5α	Host for general cloning	New England Biolabs
*E. coli* ET12567/pUZ8002	Methylation-deficient donor strain for conjugal transfer between *E. coli* and *Streptomyces*	Kieser et al., 2000
*Streptomyces* sp. SNJ042	Wild-type ohmyungsamycin-producing strain	Um et al., 2013
*ohmA*-A2 Mut1	Gly299Trp mutation in OhmA-A2 domain of SNJ042	This work
*ohmA*-A2 Mut2	Ala322Gly mutation in OhmA-A2 domain of SNJ042	This work
*ohmA*-A2 Mut3	Gly299Trp and Ala322Gly mutation in OhmA-A2 domain of SNJ042	This work
**Plasmid**		
pGEM T-Easy	*E. coli* vector for general cloning, *Amp*^R^	Promega
pKC1139	Temperature-sensitive *E. coli*–*Streptomyces* shuttle vector for gene replacement; *oriT* and *Apr*^R^	Bierman et al., 1992
pKC-Mut1	pKC1139 derivative harboring Gly299Trp mutation in A2 domain region for gene substitution	This work
pKC-Mut2	pKC1139 derivative harboring Ala322Gly mutation in A2 domain region for gene substitution	This work
pKC-Mut3	pKC1139 derivative harboring Gly299Trp and Ala322Gly mutation in A2 domain region for gene substitution	This work

### Construction of A2-Engineered Mutant Strains

For the mutation of the substrate binding residue of the OhmA-A2 domain, recombinant plasmids were prepared via site-directed mutagenesis. Two DNA fragments encoding the left and right regions containing the Gly299 and Ala322 residues, respectively, were obtained from SNJ042 genomic DNA PCR with an *Eco*RI-*Bam*HI left fragment (forward primer, 5′-TT*GAATTC*CGACCTGATGACCGCCTACA-3′; reverse primer, 5′-TT*GGATCC*GCCGCGCGGGCGGTGCGCAGGGCCTGGA-3′) and a *Bam*HI-*Xba*I right fragment (forward primer, 5′-TT*GGATCC*GGACCTGACCCTCCTCCACGC-3′; reverse primer, 5′-AA*TCTAGA*CCAGTTCGAACCGGGTGAGGT-3′); the italicized nucleotides represent the enzyme recognition sites. A *Bam*HI site was inserted between the left and right fragments by modifying the nucleotide sequence while maintaining the amino acid sequence. Each PCR product was cloned into the pGEM-T Easy vector and sequenced to generate pA2-LA and pA2-RA to use as templates for mutagenesis. The Gly299Trp mutation was performed using the pA2-LA as a template and a specific primer pair [forward: 5′-ACCTGTGGACCGGCGGCGACAT-3′, reverse: 5′-ATGTCGCCGCCGGTCCACAGGT-3′ (the substituted sequences are underlined)], which yielded pA2-Mut1. Likewise, the Ala322Gly mutation was conducted using pA2-RA as a template coupled with another primer pair [forward: 5′-CTCCTCAACGGCTACGGCCCGA-3′, reverse: 5′-TCGGGCCGTAGCCGTTGAGGAG-3′ (the substituted sequences are underlined)], which yielded pA2-Mut2. The *Eco*RI-*Bam*HI fragment from pA2-Mut1 (Mut1) and the *Bam*HI-*Xba*I fragment from pA2-RA (RA) were then co-ligated to pKC1139 through the *Eco*RI-*Xba*I site, which yielded pKC-Mut1. Similarly, pKC-Mut2 and pKC-Mut3 were constructed by combining insert fragments LA-Mut2 and Mut1-Mut2 (Supplementary Figure 1).

The recombinant plasmids for gene substitution were introduced by conjugation from non-methylating *E. coli* donor strain ET12567/pUZ8002 to the wild-type SNJ042 strain. The exconjugants were then selected on ISP4 medium supplemented with apramycin (50 μg/mL), after which the desired A2 domain mutants (i.e., which were engineered via double-crossover homologous recombination) were confirmed through *Bam*HI-digestion and PCR amplicon sequencing.

### UPLC-qTOF-HR-MS Analysis of OMSs A and B Produced by Mutant Strains

To compare the production of OMS A and OMS B from the wild-type and A2-domain-engineered strains, the strains were cultivated in 50 mL of liquid A1 + C medium (10 g of starch, 4 g of yeast extract, 2 g of peptone, and 1 g of CaCO_3_ per liter) in a 125-mL Erlenmeyer flask and incubated for 2 days at 30°C. Next, 500 μL of seed culture was transferred into 50 mL of fresh A1 + C medium in a 125-mL Erlenmeyer flask and cultivated at 30°C with shaking at 200 r/min. After 6 days of incubation, the culture broth was extracted with two volumes of ethyl acetate and the resulting concentrated extract was dissolved in acetonitrile. The samples were then analyzed by UPLC-qTOF-HR-MS. The detailed OMS A and B detection methods are described in a previous study (Kim et al., 2019).

### Large-Scale Cultivation for Culture Condition Optimization

For the optimization of culture conditions, the culture was carried out using large-scale cultures of the wild-type strain. Specifically, the SNJ042 strain stock was inoculated in 50 mL of A1 + C medium in a 125-mL Erlenmeyer flask. After cultivation for 3 days on a rotary shaker at 200 r/min at 30°C, 5 mL of the seed culture medium was inoculated in 200 mL of A1 + C medium in a 500-mL Erlenmeyer flask. After cultivation for 3 days under the same incubation conditions, the cultured medium was transferred to 1 L of medium in 2.8-L Fernbach flasks for scale-up under the following conditions. Sea salt was added from 0 to 100% w/v in 25% increases, inoculum sizes were 10, 15, and 20 mL, and five amino acids (Trp, Thr, Phe, Leu, and Val) were added alone at 1 g/L and in combination at 5 g/L. Each culture was incubated for 6 days at 170 r/min and 30°C. 5 mL of the cultured medium was then extracted and prepared for analysis as described above.

### Quantitative Analysis of OMSs

The extracts obtained from the large-scale cultures were analyzed via chromatography using an analytical reversed-phase HPLC column (Luna C18, 5 μm, 100 × 4.60 mm) with gradient conditions (42–51% MeCN-H_2_O, 0.1% trifluoroacetic acid, UV detection at 210 nm, flow rate: 0.7 mL/min) to quantitate OMSs. For quantitative HPLC analysis, purified OMS A was used as an external standard. Standard solutions containing 0.5–0.03125 mg/mL of OMS A were prepared via serial dilutions and subjected to HPLC. A linear relationship was observed between the peak area and concentration. The titers of OMSs were averaged based on three separate cultures and extractions.

## Results and Discussion

### OMS Production of A2-Domain-Engineered Strains

To engineer the substrate preference of the A2 domain toward L-Val over L-Ile, we constructed two single amino acid substituted mutants, Gly299Trp and Ala322Gly, as well as a double mutant containing both amino acid substitutions, generating SNJ042 *ohmA*-A2 Mut1, Mut2, and Mut3, respectively, based on the analysis of the specificity-conferring residues of A domains (Table 1).

**FIGURE 2:**
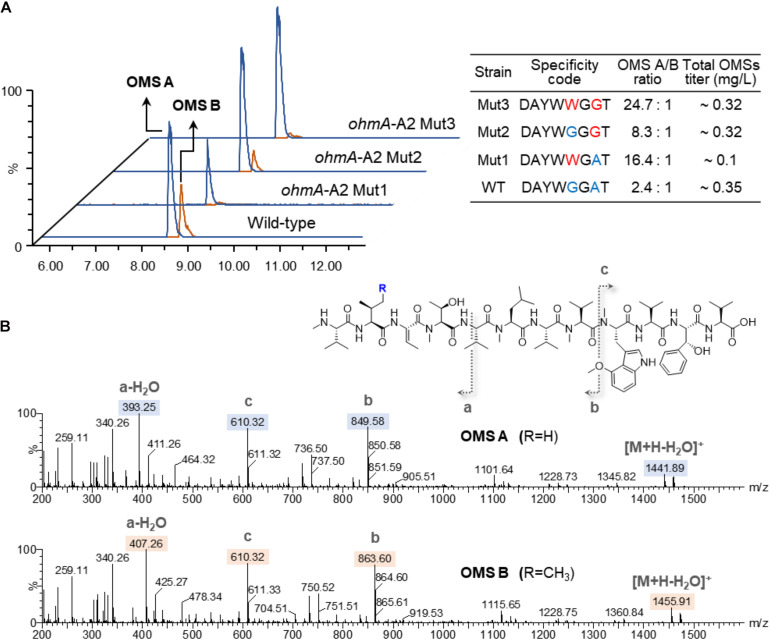
Production of OMS A and OMS B from wild-type and A2-engineered strains. **(A)** UPLC-qTOF-HR-MS analysis of the OMS A and OMS B and specificity-conferring code with OMS productivities in each strain. Chromatograms are shown for selected *m/z* values of OMS A ([M + H]^+^ = 1458.89) and OMS B ([M + H]^+^ = 1472.91). **(B)** MS/MS fragmentation patterns of OMS A and OMS B.

The OMS production of the wild-type and A2-engineered strains cultured for 6 days was analyzed by UPLC-qTOF-HRMS. OMS A and OMS B were separately eluted at 8.6 and 8.9 min retention times with *m/z* = 1458.89 and *m/z* = 1472.91, respectively (Figure 2A). Each compound was characterized by MS/MS fragmentation patterns (Figure 2B). The average production ratio of OMS A to OMS B in the wild-type strain was 2.4:1 with an OMS A yield of approximately 0.25 mg/L (Figure 2A). The total OMS titer was approximately 0.35 mg/L.

Remarkable changes in OMS production were observed in the SNJ042 *ohmA*-A2 mutant strains. OMS B production was significantly decreased in the Mut1 (Gly299Trp) strain and the OMS A/OMS B ratio shifted to 16.4:1. However, the OMS A titer in this strain was decreased by 2.6-fold (less than 0.1 mg/L) compared with that of the parental strain (Figure 2A). Furthermore, the Ala322Gly mutation (Mut2 strain) led to a 1.1-fold increase and a 3.1-fold decrease in OMS A and OMS B titers, respectively, resulting in an 8.3:1 ratio of OMS A to OMS B. Total OMS production was slightly reduced compared to the parental strain (Figure 2A). In the case of both amino acid alterations (Gly299Trp and Ala322Gly, Mut3), a drastic decrease in OMS B production with a 1.2-fold increase in OMS A was observed and the production ratio was approximately 24:1 of OMS A to B. The OMS A titer of this Mut3 strain (0.28 mg/L) was similar to that of the Mut2 stain (Figure 2A). Therefore, we confirmed that a double mutation in the SNJ042 *ohmA*-A2 Mut3 stain was most effective at increasing OMS A production while decreasing OMS B production.

The substrate specificity code of the A domain determined from the X-ray structure of the GrsA-A domain (PheA) was highly conserved across the binding pocket of the A domains and 10 specificity-conferring residues could be classified into three subgroups (Conti et al., 1997; Stachelhaus et al., 1999). The invariant Lys517 and Asp235 residues are known to interact with the carboxy and amino group of substrate amino acids, respectively. The moderately variant residues in positions 236, 301, and 330 are amino acids with hydrophobic side chains, which may not be involved in distinguishing the different substrates. Highly variant residues located at positions 239, 278, 299, 322, and 311 exhibited the highest variability depending on the amino acid substrates (Stachelhaus et al., 1999). Mutation of Gly to Trp at position 299 resulted in more substantial changes in the OMS A/OMS B ratio than Ala to Gly mutation at position 322. Based on these observations, we speculated that the mutation in position 299 would have a more profound effect on the substrate specificity than position 322. However, this Gly299Trp mutation significantly reduced OMS production. On the other hand, both mutations in positions 299 and 322 led to significant changes in the substrate preference of the A2 domain toward L-Val without decreases in OMS A production compared to the sole mutation. There are many previous reports on the engineering of the substrate specificity of A domains using a similar strategy to that reported herein. For example, the A domain in the third module of fusaricidin synthetase (FusA), which can recruit five amino acids to produce mixed fusaricidin analogs, was reprogrammed to become less promiscuous by site-directed mutagenesis, generating a mutant strain that produces a more potent L-Phe-containing congener more effectively (Han et al., 2012). However, further structural biology studies are needed to comprehensively characterize how mutations in each substrate-conferring amino acid affect the specificity and productivity of A domains.

### Effect of Sea Salt Concentration, Inoculum Size, and Amino Acid Supplementation on OMS A Production

**FIGURE 3:**
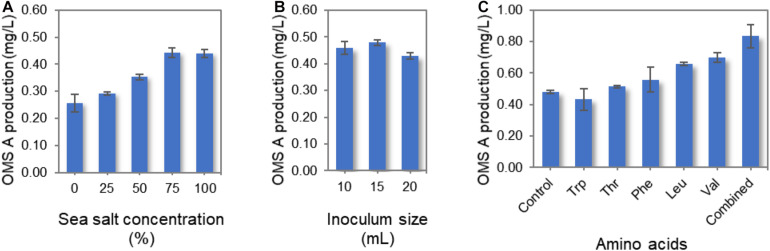
Optimization of culture conditions for OMS A production in the wild-type SNJ042 strain. **(A)** Effect of sea salt concentration on OMS A titer. **(B)** Effect of inoculum size on OMS A titer. **(C)** Effect of amino acid supplementation on OMS A titer.

Several culture conditions were optimized for the enhanced production of OMS A using the wild-type SNJ042 strain. Since SNJ042 is a marine-derived strain, the sea salt concentration in the culture medium was modified to mimic seawater. When wild-type SNJ042 was cultivated in A1 + C medium with 0, 25, 50, 75, and 100% of sea salt concentration, the highest OMS A production was observed at 75% (Figure 3A). Additionally, inoculum size is a simple but one of most critical factors for improving the titer of secondary metabolites such as NRP. In order to monitor OMS A production by inoculum size, 10, 15, and 20 mL of SNJ042 cultured seed medium were inoculated in 1 L medium with 75% of sea salt. The OMS A titer reached a maximum at a 15 mL of inoculation volume (Figure 3B).

In a previous report, the production of ecumicin, which is structurally very similar to OMS, was increased by the addition of amino acids to the culture medium (Jin et al., 2016). We also investigated the effects of varying the amino acid supply (Trp, Thr, Phe, Leu, and Val) on OMS A production, as amino acids act as protein building blocks. The wild-type strain was cultivated in A1 + C medium containing 1 g/L of each amino acid and a combination of all amino acids (1 g/L of each amino acid, total 5 g/L) under the conditions described above. Among the five amino acids, Val was found to be largely responsible for the highest OMS A production, as this amino acid is most frequently used as a biosynthetic precursor for OMSs. This observation is similar to the results obtained with ecumicin production (Jin et al., 2016). However, the mechanisms by which other amino acids (which are used once or twice during OMS assembly) affect OMS A production remain unknown. These different effects are not likely mediated by changes in growth rate, as neither amino acid supplemented on its own led to significant changes in the growth rate or morphology of the producing strain. The supplementation of all five amino acids produced the highest amount of OMS A (Figure 3C). However, increasing total amino acid supplementation beyond 5 g/L decreased OMS A production.

**FIGURE 4:**
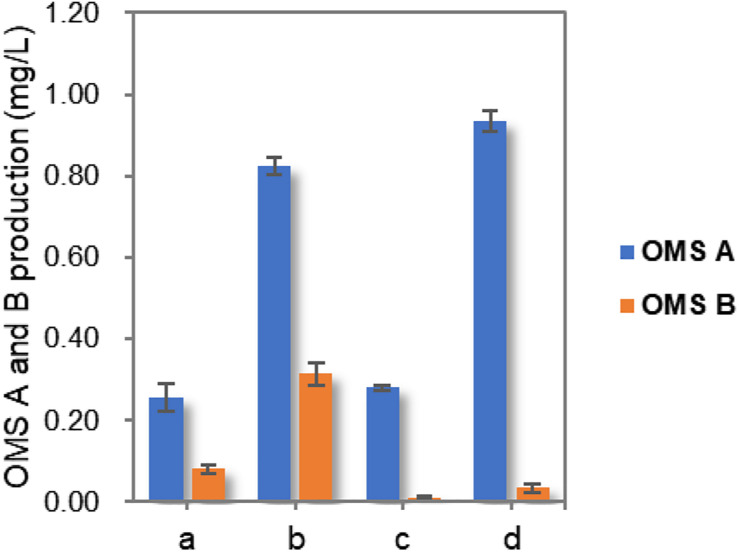
Comparison of OMS A and OMS B production in wild-type and Mut3 strains under initial condition as well as optimized culture conditions. a, wild-type strain cultured in initial conditions; b, wild-type strain cultured in optimized conditions; c, Mut3 strain cultured in the initial conditions; d, Mut3 strain cultured in optimized conditions.

Finally, the production of OMSs was compared by culturing the wild-type and *ohmA*-A2 Mut3 strains under initial and optimized culture conditions. Although the total OMS titer in Mut3 decreased slightly compared to that of the wild-type strain, OMS A production increased by up to approximately 3.8-fold (0.94 mg/L) compared to that of the wild-type strain cultured in the initial conditions (Figure 4). Importantly, only trace amounts of OMS B were produced (8.4-fold decrease compared to that of the wild-type strain cultured in the initial conditions), which enables more efficient production and purification of OMS A from the structurally similar OMS B. Although only three simple parameters were optimized in this study, more detailed optimization of the culture conditions and medium composition using a systematic method such as response surface methodology will further improve the production of OMS A.

## Conclusion

Our study demonstrated that the mutation of the specificity-conferring residues in the promiscuous A domain resulted in the predominant production of the desired product OMS A. Additionally, the optimized culture conditions, especially amino acid supplementation, in the wild-type strain resulted in the enhanced production of OMSs. The application of the optimized culture conditions to the A-domain-engineered mutant successfully increased OMS A synthesis with a dramatic decrease in OMS B production, the latter of which is a far less desirable product. These results demonstrate that a combination of substrate specificity manipulation of the A domain and the simple optimization of culture conditions can be a useful strategy to improve the yields of the desired NRP product and can be applied for many other cases where similar NRP congeners are synthesized by a single NRPS.

## Data Availability Statement

The original contributions presented in the study are included in the article/Supplementary Material. Further inquiries can be directed to the corresponding author/s.

## Author Contributions

EK, YD, YB, D-CO, and YY conceived and designed the experiments. EK, YD, and Y-HS performed the experiments. EK and YD wrote the draft manuscript. D-CO and YY reviewed and edited the manuscript. All authors contributed to the article and approved the submitted version.

## Conflict of Interest

Provisional patent applications covering this work have been filed. The authors declare that the research was conducted in the absence of any commercial or financial relationships that could be construed as a potential conflict of interest.
