# Supplementary figures and images for: Enhanced Ohmyungsamycin A Production via Adenylation Domain Engineering and Optimization of Culture Conditions
Source: Front Microbiol. 2021 Feb 17;12:626881. doi: 10.3389/fmicb.2021.626881 (PMC7925391; doi:10.3389/fmicb.2021.626881)

**Supplementary Figure S1.** A schematic diagram of cloning strategy for pKC-Mut1, pKC-Mut2, and pKC-Mut3.

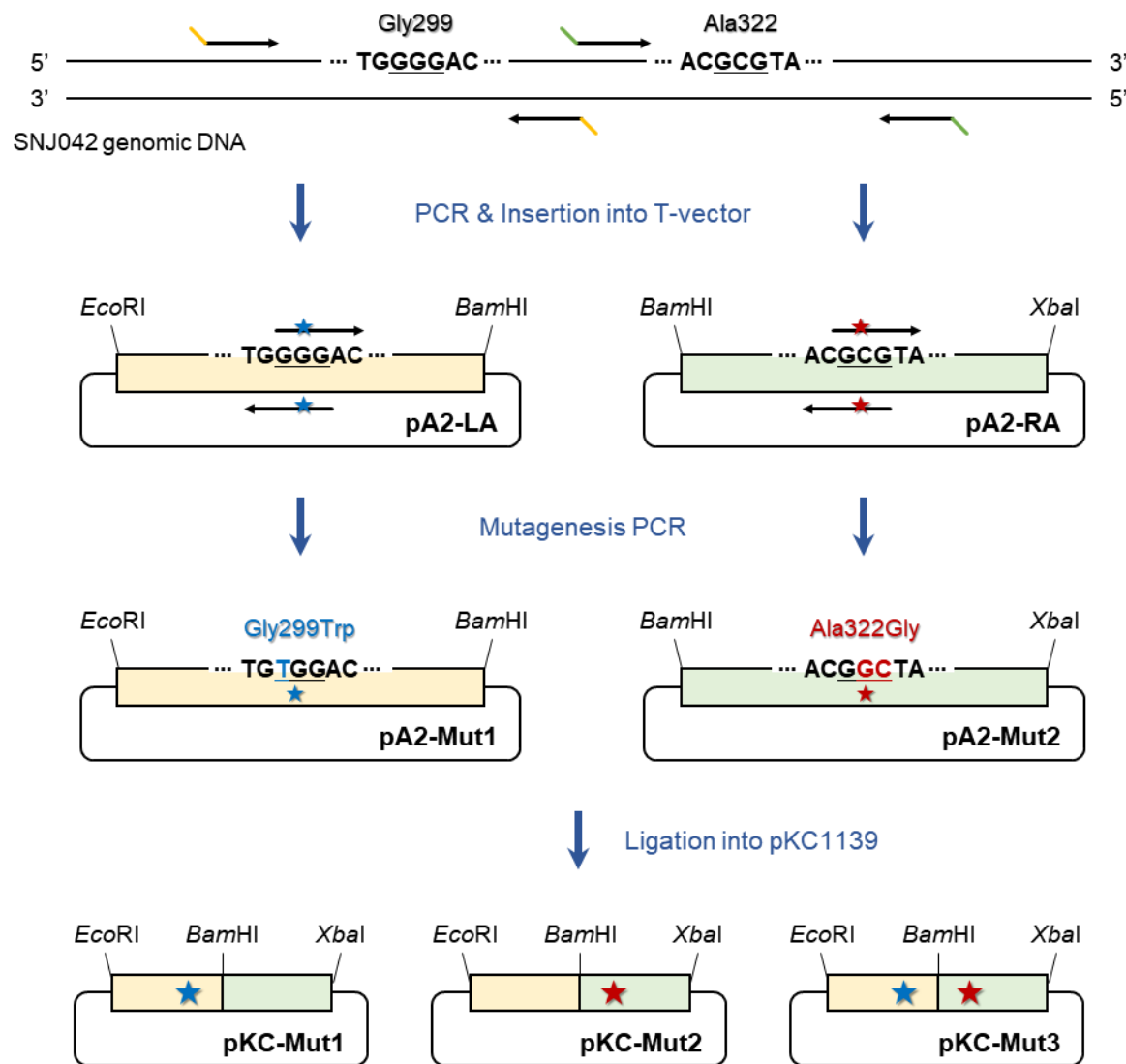

Supplement: Supplementary file 1 [file Data_Sheet_1.PDF]
